# *ST6GALNAC5* Expression Decreases the Interactions between Breast Cancer Cells and the Human Blood-Brain Barrier

**DOI:** 10.3390/ijms17081309

**Published:** 2016-08-11

**Authors:** Aurore Drolez, Elodie Vandenhaute, Clément Philippe Delannoy, Justine Hélène Dewald, Fabien Gosselet, Romeo Cecchelli, Sylvain Julien, Marie-Pierre Dehouck, Philippe Delannoy, Caroline Mysiorek

**Affiliations:** 1 Université d’Artois (UArtois), EA2465, Laboratoire de la Barrière Hémato-Encéphalique (LBHE), Lens F-62300, France; aurore.drolez@univ-artois.fr (A.D.); elodie.vandenhaute@univ-artois.fr (E.V.); fabien.gosselet@univ-artois.fr (F.G.); romeo.cecchelli@univ-artois.fr (R.C.); mpierre.dehouck@univ-artois.fr (M.-P.D.); 2 Structural and Functional Glycobiology Unit, Unité Mixte de Recherche (UMR) du Centre National de la Recherche Scientifique (CNRS) 8576, University of Lille, Villeneuve d’Ascq F-59655, France; cl-ment.delannoy@etudiant.univ-lille1.fr (C.P.D.); justine.dewald@etudiant.univ-lille1.fr (J.H.D.); philippe.delannoy@univ-lille1.fr (P.D.); 3 Cell Plasticity and Cancer, U908 INSERM, University of Lille, Villeneuve d’Ascq F-59655, France; sylvain.julien@univ-lille1.fr

**Keywords:** breast cancer, blood-brain barrier, gangliosides, G_D1α_, *ST6GALNAC5*, sialyltransferase, brain metastasis

## Abstract

The *ST6GALNAC5* gene that encodes an α2,6-sialyltransferase involved in the biosynthesis of α-series gangliosides, was previously identified as one of the genes that mediate breast cancer metastasis to the brain. We have shown that the expression of *ST6GALNAC5* in MDA-MB-231 breast cancer cells resulted in the expression of G_D1α_ ganglioside at the cell surface. By using a human blood-brain barrier in vitro model recently developed, consisting in CD34^+^ derived endothelial cells co-cultivated with pericytes, we show that *ST6GALNAC5* expression decreased the interactions between the breast cancer cells and the human blood-brain barrier.

## 1. Introduction

The modification of cell surface glycosylation is one of the most important phenotypic rearrangements that occur during carcinogenesis. It mainly affects the terminal part of the carbohydrate moiety of glycoproteins and glycolipids, leading to the expression of tumor-associated carbohydrate antigens (TACA). Most TACAs are sialylated and changes in sialylation were clearly demonstrated to affect cellular recognition, cell adhesion, and signaling and, consequently, the cell’s behavior. Gangliosides are glycosphingolipids (GSLs) carrying one or several sialic acid residues. They are essentially located on the outer leaflet of the plasma membrane where they can interact with transmembrane receptors or signal transducers involved in cell proliferation, adhesion, and motility. In adult, complex gangliosides from b- and c-series are normally restricted to the nervous system but a re-expression of complex gangliosides is observed in a variety of cancers including neuro-ectoderm-derived cancers, non-small cell lung carcinoma, and breast cancer [1]. In particular, G_D3_ and G_D2_ are considered as melanoma- and neuroblastoma-associated antigens playing a key role in tumor development, and are used as targets for cancer immunotherapy [2]. However, the mechanisms by which tumor-associated gangliosides induce invasive and metastatic phenotypes of tumor cells remain to be clarified.

α-Series gangliosides define a particular sub-class of GSLs containing Neu5Ac α2,6 linked to the GalNAc residue of the gangliopentaosyl backbone Neu5Acα2-3Galβ1-3GalNAcβ1-4Galβ1-4Glc (IV^3^Neu5Ac_1_Gg_4_). The typical α-series ganglioside G_D1α_ (IV^3^Neu5Ac_1_,III^6^Neu5Ac_1_Gg_4_-Cer) was first isolated as a minor compound from rat hepatoma cells [3] and from bovine brains [4], with an expression restricted to particular cell populations of the brain and cerebellum [5]. Three members of the CMP-Neu5Ac: β-*N*-acetylgalactosaminide α2,6-sialyltransferase family (ST6GalNAc III, V, and VI) were shown to catalyze in vitro the transfer of a sialic acid residue onto G_M1b_ (IV^3^Neu5Ac_1_Gg_4_-Cer) to form G_D1α_ [6]. However, according to its substrate specificity and expression pattern, ST6GalNAc V is considered as the main G_D1α_ synthase. *ST6GALNAC5* cDNA was cloned from mouse brains [7,8] and the *st6galnac5* gene is specifically expressed in mouse brain tissues, mostly in the forebrain and cerebellum [8]. When expressed as a soluble recombinant protein, the mouse ST6GalNAc V showed α2,6-sialyltransferase activity almost exclusively for G_M1b_, while being inactive toward glycoproteins [7]. The enzymatic activity of human ST6GalNAc V was never investigated in detail, but we have recently shown that transfection of human ST6GalNAc V cDNA into MDA-MB-231 breast cancer cells resulted in the expression of G_D1α_ at the cell surface [9].

To date, the specific function of α-series gangliosides is poorly understood. It has been proposed that G_D1α_ could play a role in Purkinje cell functions in the cerebellum [5] and that G_D1α_ could serve as an adhesion molecule for high-metastatic murine lymphosarcoma cells in the adhesion to hepatic endothelial cells [10]. Recently, *ST6GALNAC5* was identified as one of the genes over-expressed in breast cancer cell populations selected for their ability to produce brain metastases [11]. ShRNA inhibition of *ST6GALNAC5* expression reduced the capacity of breast cancer cells to produce brain metastases, whereas the expression of *ST6GALNAC5* in parental cell lines promoted brain metastases formation [11]. Moreover, *ST6GALNAC5* was shown to improve the capacity of breast cancer cells to transmigrate across a human umbilical vein endothelial cells (HUVECs) in vitro model of the blood-brain barrier [11].

The blood-brain barrier (BBB), localized at the level of brain capillary endothelial cells (ECs), controls and restricts the exchanges between the blood and the brain tissues. The BBB presents a specific architecture where the capillary ECs share a split basement membrane with pericytes and are surrounded together by astrocyte end-feet. The BBB forms with pericytes, neurons, glial cells, and the extracellular matrix, the neurovascular unit (NVU). The interplays and communications between the different components of NVU allow the BBB-specific differentiation of ECs, which exhibit a network of tight junctions, express efflux pumps and specific receptors and transporters. These specific and restrictive properties control and limit the access to the brain parenchyma of many cells and substances. During the last decades, most in vitro BBB models were developed using animal cells (mouse, rat, bovine, pig) isolated from brain microvessels as the primary culture or immortalized [12], whereas human culture models commonly use HUVECs, which display only a limited tightness and not a BBB phenotype. 

In vitro approaches are required to identify cellular and molecular interactions between cancer cells and BBB endothelium. However, while numerous studies were performed with in vitro models, the heterogeneity and the quality of BBB models used is a limitation to the extrapolation of results to in vivo context, showing that the choice of a model that fulfills the properties of human BBB is essential.

In that context, we recently developed a human BBB in vitro model consisting in CD34^+^ hematopoietic stem cells derived endothelial cells co-cultivated with brain pericytes [13,14] and displaying improved BBB properties closed to those observed in vivo. The model proved valuable in the study of cancer cells tropism as the adhesion and transmigration capacities of breast cancer cells were found to be in accordance with the cancer cell molecular subtypes, fitting well with their propensity to form brain metastases [15,16]. We have used this CD34^+^ derived human BBB model to investigate the role of G_D1α_ in adhesion and transmigration of breast cancer cells and contrary to what was observed in a HUVECs in vitro model, *ST6GALNAC5* cDNA expression resulted in a decrease of the interactions between MDA-MB-231 breast cancer cells and the CD34^+^ derived human BBB model.

## 2. Results

### 2.1. Brain Targeting Cells Interaction Analysis on the Human in Vitro Blood-Brain Barrier (BBB) Model

In order to investigate the mechanisms of brain tropism during the initial steps of breast cancer brain metastases formation, the interactions of breast cancer cells with the BBB were analyzed using an in vitro approach. For this purpose, adhesion and transmigration assays of brain-targeting breast cancer cells were performed on a human BBB in vitro model named Brain-Like endothelial Cells (BLECs) that we recently developed [13,14]. The BLECs model consists of endothelial cells derived from CD34^+^ hematopoietic stem cells co-cultivated with brain pericytes. The BLECs model displays improved BBB properties close to those observed in vivo, such as low permeability to the BBB integrity marker, continuous localization at the cell border of tight junction proteins (Claudin-5, occludin, ZO-1), and expression of functional efflux pumps (P-gP, BCRP) [13,14].

**Figure 1:**
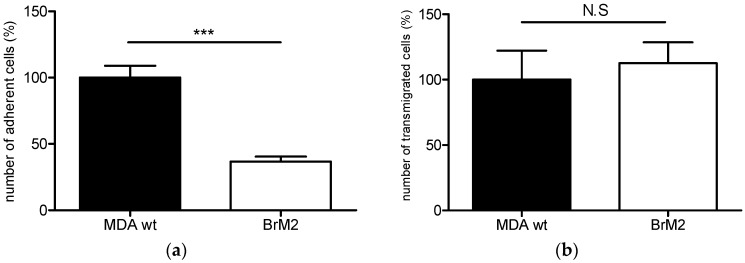
Adhesion (**a**); and transmigration (**b**) of MDA-MB-231 wild type (wt) and BrM2 breast cancer cell lines on the human BLECs model. The number of adherent or transmigrated MDA-MB-231 wt cells was set up to 100% and equal to, respectively, 579 and 98 cells. Results are the mean in triplicate, and representative of two independent experiments N.S: not significant; *** *p* < 0.001.

The adhesion and transmigration of MDA-MB 231 BrM2 cell line (BrM2) was compared to the parental cell line MDA-MB-231 wild type (wt). The BrM2 cell line was previously generated by two rounds of in vivo selection in mice, and showed a significant increase in brain metastases formation [11]. As shown in Figure 1a, after two hours of incubation, the adhesion rate of BrM2 on the BBB ECs was 63.4% lower than the parental cells and no increase in BBB permeability to lucifer yellow (LY) was observed for MDA and BrM2 (0.57 ± 0.08 × 10^−3^ cm·min^−1^ and 0.66 ± 0.1 × 10^−3^ cm·min^−1^, respectively) compared to the control condition before adhesion (permeability coefficient (Pe) = 0.58 ± 0.07 × 10^−3^ cm·min^−1^). As the adhesion of breast cancer cells on the BBB ECs is required, but not enough to reach the brain parenchyma, the transmigration was quantified and it was revealed that the BrM2 transmigrate at the same rate compared to the parental cell line MDA-MB-231 wt (Figure 1b). No increase in BBB permeability to LY was measured following transmigration of MDA and BrM2 (0.73 ± 0.03 × 10^−3^ cm·min^−1^ and 0.78 ± 0.01 × 10^−3^ cm·min^−1^, respectively) compared to the control condition without transmigration.

### 2.2. Molecular Characterization of MDA-MB-231 BrM2 Cells

**Figure 2:**
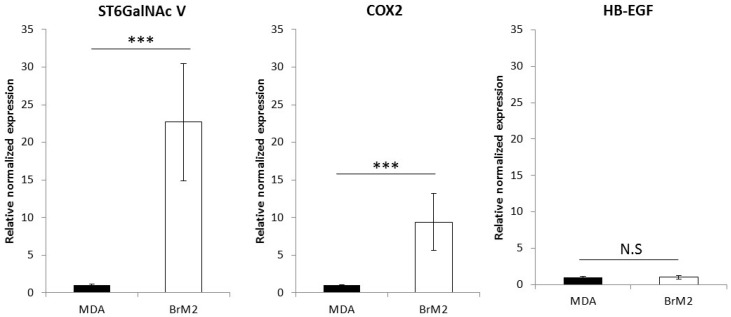
qPCR analysis of *COX2*, *HB-EGF*, and *ST6GALNAC5* in MDA-MB-231 wt and BrM2. Quantification was performed by the method described by Pfaffl [17] and normalized to Actin. N.S: not significant; *** *p* < 0.001.

The BrM2 cell line was previously described to over-express a set of genes potentially involved in brain metastasis, including *COX2*, *HBEGF* and *ST6GALNAC5* [11]. The expression of these genes was quantified by qPCR. As shown in Figure 2, a 23-fold increased expression of *ST6GALNAC5* and 10-fold increase of *COX2* were measured in BrM2 compared to parental MDA-MB-231 wt. However, no difference of expression was measured for *HBEGF* in BrM2 compared to parental MDA-MB-231 wt.

*ST6GALNAC5* gene encodes a GalNAc α2,6-sialyltransferase involved in the biosynthesis of α-series gangliosides, mainly G_D1α_. According to the fact that G_D1α_ could serve as an adhesion molecule for breast cancer cells in the adhesion to BBB endothelial cells and promote brain metastasis, the GSL composition was analyzed to determine the impact of the increased *ST6GALNAC5* expression on the glycosylation of MDA-MB-231 BrM2 cells. Total GSLs were extracted from MDA-MB-231 wt and BrM2, purified by reverse phase chromatography and permethylated prior to Matrix assisted laser desorption-ionization-mass spectrometry (MALDI-MS) analysis.

**Figure 3:**
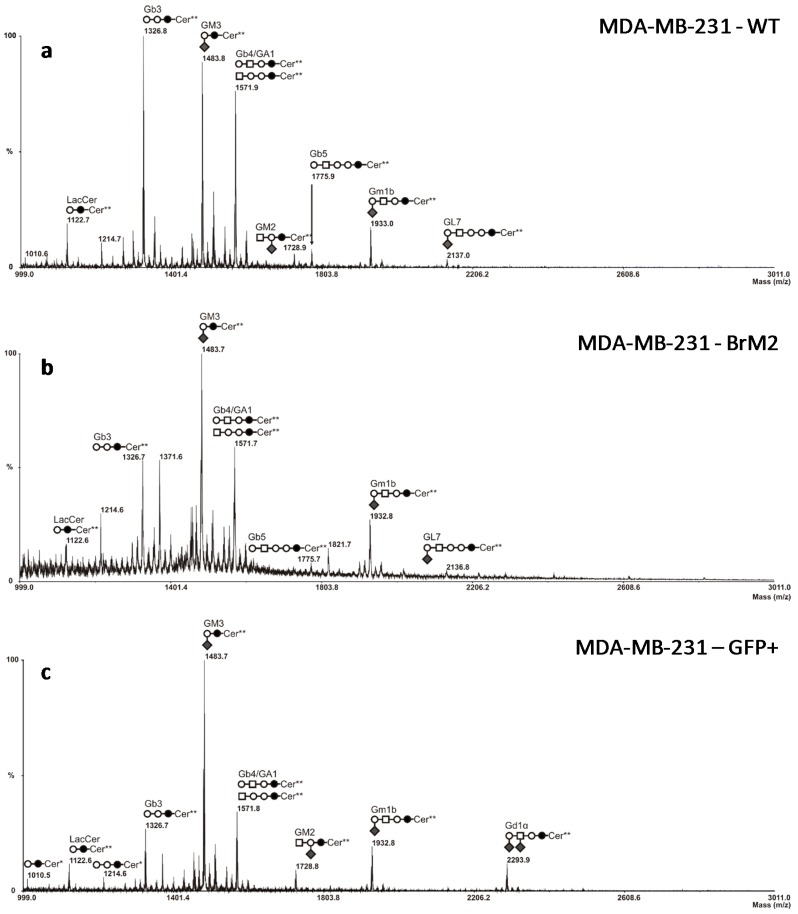
Comparison of mass spectrometry (MS) profiles of permethylated glycosphingolipids purified from MDA-MB-231 wt (**a**); BrM2 (**b**); and green fluorescent protein positive (GFP+) (**c**) cell population. Glycosphingolipids (GSL) are present as d18:1/C16:0 (Cer*) and d18:1/C24:0 (Cer**) isomers. 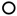, Gal; 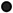, Glc; 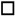, GalNAc; 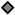, Neu5Ac.

As previously shown [18] MDA-MB-231 wt expresses neutral globosides G_b3_ and G_b4_ and monosialylated gangliosides, mainly G_M3_ (Figure 3a). The precursor lactosylceramide (LacCer) was also detected, as well as a monosialoganglioside at *m*/*z* 1933, which was confirmed to correspond to G_M1b_ by matrix assisted laser desorption-ionization time-of-flight (MALDI-TOF)/TOF fragmentation analysis (data not shown). Two ceramide isoforms are commonly expressed in human tissues due to the substitution of the sphingosine moiety by palmitic acid C16:0 (Cer*) or lignoceric acid C24:0 (Cer**). As shown in Figure 3b, the composition in GSLs of BrM2 cells was similar to wt cells and no expression of G_D1α_ was detected as indicated by the absence of signal at *m*/*z* 2293.9, which was identified in MDA-MB-231 green fluorescent protein positive (GFP+) cell population (Figure 3c) by MALDI-TOF/TOF fragmentation analysis (Figure S1). 

### 2.3. Involvement of G_D1α_ Over-Expression in Interaction Processes of Breast Cancer Cell Lines with the BBB

**Figure 4:**
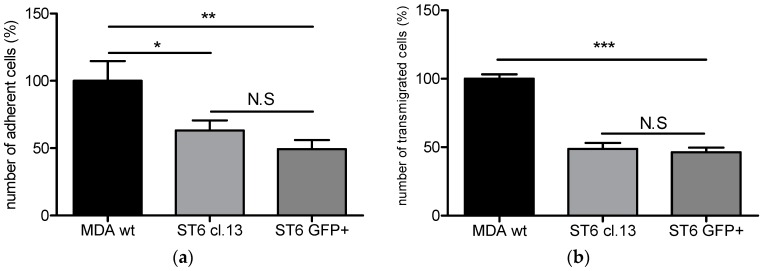
Adhesion (**a**); and transmigration (**b**) of MDA-MB-231wt, Clone #13 (ST6 cl. 13), and GFP+ breast cancer cell population (ST6 GFP+) on the human Brain-Like endothelial Cells (BLECs) model. The number of adherent or transmigrated MDA-MB-231 wt cells was set up to 100% and equal to, respectively, 533 and 117 cells. Results are the mean in triplicate, and representative of two or three independent experiments. N.S: not significant; * *p* < 0.01; ** *p* < 0.005; *** *p* < 0.001.

In order to specifically identify the effect of *ST6GALNAC5* over-expression on the adhesion and transmigration of breast cancer cells, two cell populations were generated, Clone #13 and a polyclonal GFP-positive cell population, in which *ST6GALNAC5* cDNA was 10-fold and 60-fold over-expressed compared to control MDA-MB 231 wt, respectively [9]. These two cell populations were previously demonstrated to express G_D1α_ ganglioside by MALDI-TOF/TOF fragmentation analysis (Figure S1). The adhesion and transmigration capacities of these two cell populations were determined using the human BBB in vitro model. As shown in Figure 4a, the results obtained were similar to those obtained with BrM2 cells as adhesion of the Clone #13 and GFP+ cell population were 40% and 50% decreased compared to MDA-MB-231 wt, respectively. A 55% and 50% decrease of transmigration rate was also observed for Clone #13 and the GFP+ cell population, respectively (Figure 4b). Following adhesion and transmigration assays, no increase in BBB permeability was measured for Clone #13 and GFP+ cell populations (0.87 ± 0.03 × 10^−3^ cm·min^−1^ and 0.92 ± 0.07 × 10^−3^ cm·min^−1^ respectively) compared to the control condition before transmigration (Pe = 0.90 ± 0.04 × 10^−3^ cm·min^−1^).

### 2.4. Does the Specificity of Interactions Depend on Cells Species?

**Figure 5:**
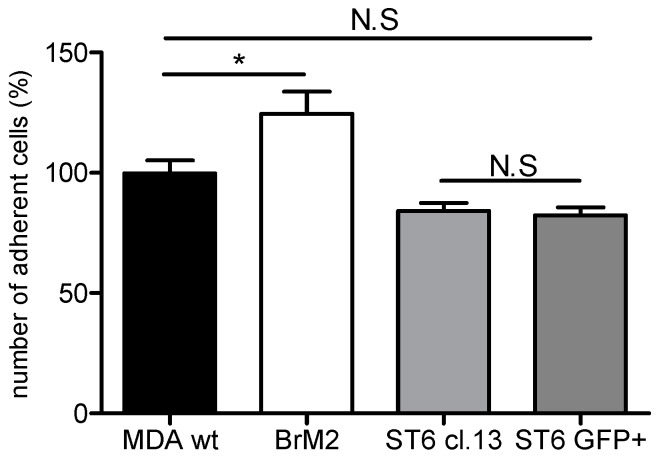
Adhesion of MDA-MB-231 wt, BrM2, Clone #13, and GFP+ breast cancer cell populations on a mouse blood-brain barrier (BBB) model. The number of adherent MDA-MB-231 wt cells was set up to 100% and equal to 286 cells. Results are the mean in triplicate, and representative of two independent experiments. N.S: not significant; * *p* < 0.1.

As the BrM2 cells were generated and selected according to their increased brain metastatic activity in immunodeficient mice, a mouse-specific molecular mechanism of interaction could take place between human breast cancer cells and murine brain endothelial cells. Hence, in order to determine if the capacity of interaction could be modified according to the species, we measured adhesion of breast cancer cells on a mouse BBB in vitro model. As shown in Figure 5, after two hours of incubation, a significant 25% increase in adhesion was observed for BrM2 cells compared to control MDA-MB-231 wt. However, no increase of adhesion was measured for ST6GalNAc V over-expressing cells, Clone #13, and GFP+ cell population. Following adhesion assay of cancer cell populations, no increase in BBB permeability was measured (0.47 ± 0.1 × 10^−3^ cm·min^−1^) compared to the control condition before adhesion (Pe = 0.36 ± 0.12 × 10^−3^ cm·min^−1^).

## 3. Discussion

With the improvement of cancer therapeutic strategies to treat systemic disease, the incidence of brain metastases has increased. Brain metastases (BM) represent the most frequent intracranial tumors in adults. Breast cancer is after lung cancer, the second type of cancer which has the highest incidence to develop metastasis in the brain; about 30% of women with breast cancer developing metastases to the brain. To form metastases in distant organs, cancer cells from the primary tumor have to successfully achieve the multistep process of metastatic cascade that includes the escape from the primary tumor, the survival in the circulation, the interactions with the vascular wall of the targeted organ, the extravasation through the endothelial cell layer and finally the adaptation to the host environment.

In the case of brain metastases, the cancer cells also have to interact and cross the highly restrictive and specific BBB, localized at the level of brain capillary endothelial cells. This barrier maintains the brain homeostasis thanks to specific properties that limit the access to the brain parenchyma. In the therapeutic strategy against BM, besides the surgical resection and radiotherapy, the chemotherapy plays an underlying role with a limited efficacy mainly attributed to the BBB. In this context, the understanding the biology is prime of important for both the prediction of patients with high risk to develop BM and the discovery of new drug targets [19].

For breast cancer patients, the risk to develop BM is associated with the breast cancer molecular subtypes, which are described to have different clinical behaviors. In this context, analyses of cellular and molecular events are performed during the metastatic cascade in order to better understand the behavior of breast cancer cells and to identify a molecular signature that could predict the risk of brain metastases. The widely used experimental strategy consists of the transcriptional profiling of cancer cells following multiple cycles of injection in rodents and a comparison with the molecular profile obtained from brain metastases clinical samples. This experimental procedure was used by Bos et al. [11] in order to generate a brain targeting breast cancer cell line named BrM2, deriving from the triple negative breast cancer cell line MDA-MB-231. The gene encoding the ganglioside specific sialyltransferase ST6GalNAc V was one of the genes whose expression was upregulated in BrM2 and was identified as a potential mediator for cancer cells transmigration through an endothelial in vitro barrier model using HUVECs. Here, using our well-characterized human BBB in vitro model, we clearly demonstrate a decreased adhesion and no change in transmigration of BrM2 cells compared to the control. These results are not line with the data from Bos et al. who previously showed that the expression of *ST6GALNAC5* cDNA was sufficient to increase the transmigration activity of MDA-MB-231 BrM2 cells through the HUVECs BBB in vitro model [11]. 

As a matter of fact, the phenotype of endothelial cells used in the in vitro approach is of prime importance to study cellular and molecular interactions occurring at the level of the BBB, in order to specifically identify the mechanisms occurring at this particular interface as previously described [15]. Hence, the use of endothelial cells without a validated BBB phenotype, can generate data that cannot be correlated with the in vivo situation. 

In vitro, ST6GalNAc V is known to catalyze the transfer of a sialic acid residue onto G_M1b_ to form G_D1α_ [6] and is considered as the main enzyme for the biosynthesis of α-series gangliosides, the expression of which being normally restricted to the brain [5]. Interestingly, it has been recently shown that G_D1α_ could serve as an adhesion molecule for highly-metastatic murine lymphosarcoma cells in the adhesion to hepatic endothelial cells [10]. Similarly, the expression of G_D1α_ at the cell surface of breast cancer cells could improve their capacity to interact with brain capillary endothelial cells. We also used a cell line deriving from parental MDA-MB-231 by transfection of human *ST6GALNAC5* cDNA and over-expressing ST6GalNAc V. Our experiments have revealed that the over-expression of the active form of ST6GalNAc V, associated with the expression of G_D1α_ at the cell surface, displays a reduced capacity of interaction and transmigration in the human BBB in vitro model. These results were similar to those we obtained with BrM2 cells indicating that the over-expression of ST6GalNAc V decreased the amount of breast cancer cells able to interact with the BBB ECs. 

Nevertheless, as *ST6GALNAC5* gene encodes a sialyltransferase involved in the biosynthesis of gangliosides and should, therefore, modify the cell surface glycosylation, mass spectrometry analysis was performed on BrM2 cells in order to identify a change in GSLs composition potentially involved in the interaction of breast cancer cells with the BBB. Surprisingly, no difference in GSLs content was observed compared to the parental MDA-MB-231 cells whereas BrM2 cells express the substrate of ST6GalNAc V. As the analysis of BrM2 glycosylation was only performed on the glycosphingolipid fraction, we cannot exclude an unusual activity of ST6GalNAc V in BrM2 cells that could sialylate *O*-glycans. However, these results allowed us to assume that the expression of *ST6GALNAC5* in BrM2 does not lead to a functional enzyme that modulates the GSLs content. At least, as the BrM2 cells were generated in mice, and considering that cancer cells can develop a host adaptation to maximize their colonizing properties, we analyzed the adhesion of BrM2 and ST6GalNAc V over-expressing cells on a mouse BBB in vitro model. Our results revealed that the adhesion of BrM2 is increased on mouse BBB endothelial cells compared to the ST6 GFP+ and Clone #13, indicating that the cells generated in mouse display an increase of interaction contrary to the cells in which *ST6GALNAC5* cDNA was transfected. Environment adaptation is a well-known mechanism and the adaptation of breast cancer cells to the murine environment could explain at least in part the increased adhesion of BrM2 cells on a mouse BBB in vitro model. However, this result is difficult to correlate with the fact that the expression of *ST6GALNAC5* cDNA in BrM2 MDA-MB-231 increased the transmigration of cells through HUVECs [11]. Hence, our experimental approach highlighted that ST6GalNAc V does not seem to be a mediator that increases breast cancer cell interaction with the human BBB. In addition, the expression of ST6GalNAc V in cancer cells is not directly correlated with the expression of its product G_D1α_ that is depending on the presence of the precursor G_M1b_. As the expression of ST6GalNAc V is normally restricted to the brain, the expression of the enzyme in cancer cells seems particularly suitable for the development of tumors in the brain parenchyma. However, it was recently shown that, using specific monoclonal antibodies reactive with G_D1α_ or G_M1b_, only a few human cancer cell lines show significant expression of these gangliosides [20]. These antibodies could be used in the near future to determine the expression of these gangliosides in breast cancer tumors, as well as the implication of G_D1α_ in the formation of breast cancer cell metastases. Moreover, molecular analysis of the transmigrated breast cancer cells, and also studies of their capacities of colony-forming, should be done in order to better characterize brain-metastatic breast cancer cells. 

## 4. Materials and Methods 

### 4.1. Human Brain-Like Endothelial Cells (BLECs) BBB in Vitro Model

The human BBB in vitro model consists of endothelial cells derived from CD34^+^ cord blood hematopoietic stem cells in co-culture with bovine brain pericytes, as described by Cecchelli et al. 2014 [13]. The collection of human umbilical cord blood requires infants’ parents signed consent form in compliance with French legislation. The protocol was approved by the French Ministry of Higher Education and Research (CODECOH Number DC2011-1321). All experiments were carried out in accordance with the approved protocol. According to the method described by Pedroso et al. [21], CD34^+^-cells are isolated from human umbilical cord blood and then differentiated into endothelial cells following exposure to Vascular Endothelial Growth Factor (VEGF from PrepoTech Inc., Rocky Hill, NJ, USA) at 50 ng/mL. 

To perform the co-culture, CD34^+^ derived endothelial cells (CD34^+^-ECs) were seeded on Matrigel™ (BD Biosciences, San Jose, CA, USA)-coated filters (Costar Transwell inserts, pore size 0.4 or 3 µm, 12-well format, Corning Inc., Corning, NY, USA) (8 × 10^4^ cells/cm^2^). After six days of culture alone without medium in the lower compartment, CD34^+^-ECs were placed above a well containing a bovine brain pericyte culture. The phenotype of the pericytes was characterized according to Vandenhaute et al. [22]. The co-culture medium, endothelial cell medium (ECM) supplemented with 5% heat inactivated fetal calf serum (FCS), and 50 µg/mL gentamicin, was changed every two days. After six days of co-culture, the model was stable and ready for experiment [13]. The co-culture system allows the delimitation of two compartments, the luminal compartment with endothelial cells (blood side) and the abluminal compartment with the pericytes (brain side). The co-culture system was described in details in Cecchelli et al. 2014 [13].

### 4.2. Murine BBB in Vitro Model

In accordance with the French legislation the animal house of the Université d’Artois obtained approval from the protecting population departmental directorate under number B62-498-5. In compliance with the new European directive (Directive 2010/63/EU), all of the procedures were submitted to the ethics committee (comité d'éthique en experimentation animale Nord—Pas-de-Calais; C2EA 75) and the French Ministry (ministère de l’enseignement supérieur et de la recherche: direction générale pour la recherche et l'innovation) for authorization, were approved and referenced under the number 2015090115412152. Mice (C57Bl6/J) were supplied by Laboratoire Janvier (Le Genest-Saint-Isle, France) and housed in a temperature-controlled pathogen-free room with light from 07:00 to 19:00 (daytime) and had free access to food and water and live in an enriched environment.

Endothelial cells were extracted from mice brain microvessels using the method described by Coisne et al. [23] and seeded on Matrigel™-coated filters (Costar Transwell 0.4 µm, 12-well format). All experiments were performed within the framework of the French legislation that controls animal experimentation. Cells were cultivated until confluence in Dulbecco’s modified Eagle medium (DMEM) medium, 5% heat-inactivated FCS, 2 mM l-glutamine, 50 µg/mL gentamicin, and 1 ng/mL basic fibroblast growth factor (bFGF). This medium was changed every day. Endothelial cells formed a confluent monolayer and were used for experiment after five days.

### 4.3. Human Breast Cancer Cell Lines Culture

Breast cancer cell lines MDA-MB-231 (HTB-26, ATCC^®^, Manassas, VA, USA), MDA-MB-231-Clone #13-, and GFP+ *ST6GALNAC5*-transfected cells [9], and MDA-MB-231 BrM2 [11] cells were cultivated in DMEM medium with 4.5 g/L d-glucose, 10% heat-inactivated FCS, 2 mM l-glutamine, and 5 µg/mL penicillin-streptomycin. Cells were cultivated for three weeks before being used in adhesion or transmigration experiment.

### 4.4. Adhesion and Transmigration Assays

Adhesion and transmigration assays were carried out as previously described [15]. Breast cancer cells were loaded with a fluorescent CellTracker before adhesion and transmigration kinetics (Invitrogen, Carlsbad, CA, USA).

After treatment with ethylenediaminetetraacetic acid (EDTA) and mechanical dissociation, cancer cells were seeded at 2 × 10^4^ or 8 × 10^4^ per filter containing EC monolayers for adhesion or transmigration assays, respectively. After 120 min (adhesion) or 16 h (transmigration) filters were fixed with 4% paraformaldhehyde solution for 10 min. After the staining of nuclei with Hoechst 33358 (BisBenzimide, MP Biochemicals, Irvine, CA, USA), the filters were mounted using Mowiol solution containing DABCO (1,4-Diazobicyclo-(2.2.2-octane)) as an anti-fading agent.

The quantification of adherent and transmigrated cancer cells was done manually on the total surface of each filter under a Leica DMR fluorescence microscope (Leica Microsystem, Wetzlar, Germany). The number of adherent or transmigrated MDA-MB-231was set to 100%. All results were expressed as the mean ± standard error of the mean (SEM) from two or more independent experiments. Statistical significance was assessed by *t*-test. All statistical analyses were performed using GraphPad Prism version 5.0 for Windows (GraphPad Software, San Diego, CA, USA).

### 4.5. BBB Permeability Measurement

The integrity of BBB ECs was evaluated by the permeability measurement according to the method described by Dehouck et al. [24] using the diffusion of hydrophilic molecules, Lucifer Yellow (LY, lucifer yellow CH dilithium salt, Sigma-Aldrich, St-Louis, MO, USA) or [14C]-saccharose (1 μCi/mL, molecular mass (MM) = 342) (Amersham Biosciences, Piscataway, NJ, USA), that cross the BBB poorly and are used as integrity markers. The protocol was performed as previously described [15]. The endothelial permeability coefficients (Pe) of the molecules are expressed in cm/min.

### 4.6. mRNA Extraction and PCR Analysis

Cancer cells were lysed with 500 μL of RLT lysis buffer (Qiagen, Les Ullis, France). mRNA was purified using the RNeasy total RNA extraction kit (Qiagen), following the manufacturer’s instructions. After this step, the mRNA’s purity and concentration were assessed by measuring the absorbance at 260, 280, and 320  nm using the Take 3 microplate reader protocol (Synergy™ H1, BioTek Instruments, Colmar, France).

For each condition, cDNAs were obtained from 0.5 μg of mRNA using iScript™ Reverse Transcription Supermix (BioRad, Marnes-la-Coquette, France), according to the manufacturer’s instructions. Real-time PCR experiments were performed using the Sso Fast EvaGreen Master Mix kit (BioRad). Primers for *ACTB* (sense: 5′-ggagcacagagcctcgcctt-3′, antisense: 5′-acatgccggagccgttgtcg-3′), *ST6GALNAC5* (sense: 5′-ggatcccaatcacccttcag-3′, antisense: 5′-tagcaagtgattctggtttcca-3′), *COX2* (sense: 5′-tccaccaacttacaatgctgac-3′, antisense: 5′-cacaggaggaagggctctagta-3′) and *HBEGF* (sense: 5′-ggttaccatggagagaggtgtc-3′, antisense: 5′-gaccagcagacagacagatgac-3′) were designed using Primer 3 software. For each primer, amplification was carried out for 40 cycles with an annealing temperature of 60 °C in a CFX96 thermocycler (BioRad). The efficiency was calculated for each primer pair (CFX Manager, BioRad). Melting curve analysis was performed after the amplification cycles, in order to check the specificity/purity of each amplification. Gene expression levels were evaluated according to the ΔΔ*C*t method and normalized against Actin expression.

### 4.7. Extraction and Preparation of Glycolipids

Twenty dishes (10 cm diameter) of cultured cells were washed twice with ice-cold PBS and cells were sonicated on ice in 200 µL of water. The resulting material was dried under vacuum and sequentially extracted by CHCl_3_/CH_3_OH (2:1, *v*/*v*), CHCl_3_/CH_3_OH (1:1, *v*/*v*) and CHCl_3_/CH_3_OH/H_2_O (1:2:0.8, *v*/*v*/*v*) using intermediary centrifugations at 2500× *g* for 20 min. Supernatants were pooled, dried and subjected to a mild saponification in 0.1 M NaOH in CHCl_3_/CH_3_OH (1:1) at 37 °C for 2 h and then evaporated to dryness [25]. Samples were reconstituted in CH_3_OH/0.1% TFA in H_2_O (1:1, *v*/*v*) and applied to a reverse phase C_18_ cartridge (Waters, Milford, MA, USA) equilibrated in the same solvent. After washing with CH_3_OH/0.1% TFA in H_2_O (1:1, *v*/*v*), GSL were eluted by CH_3_OH, CHCl_3_/CH_3_OH (1:1, *v*/*v*), and CHCl_3_/CH_3_OH (2:1, *v*/*v*). The elution fraction was dried under a nitrogen stream.

### 4.8. Mass Spectrometry Analysis of Glycosphingolipids (GSLs)

Prior to mass spectrometry analysis, GSL were permethylated according to Ciucanu and Kerek [26]. Briefly, compounds were incubated 2 h in a suspension of NaOH in dry dimethylsulfoxyde (DMSO) (400 µL) and CH_3_I (200 µL). The methylated derivatives were extracted in CHCl_3_ and washed several times with water. The reagents were evaporated and the sample was dissolved in CHCl_3_ in the appropriate dilution. MALDI-MS and MS/MS analyses of permethylated GSL were performed on a 4800 Proteomics Analyzer (Applied Biosystems, Framingham, MA, USA) mass spectrometer, operated in the positive reflectron mode. For MS acquisition, 5 µL of diluted permethylated samples in CHCl_3_ were mixed with 5 µL of 2,5-dihydroxybenzoic acid matrix solution (10 mg/mL dissolved in CHCl_3_/CH_3_OH (1:1, *v*/*v*)). The mixtures (2 µL) were then spotted on the target plate and air dried. MS survey data comprises a total of 50 sub-spectra of 3000 laser shots. Peaks observed in the MS spectra were selected for further MS/MS. Collision-induced dissociation (CID) MS/MS data comprises a total of 100 sub-spectra of 3000 laser shots. Two or more spectra can be combined post-acquisition with mass tolerance set at 0.1 Da to improve the signal-to-noise (S/N) ratio. The potential difference between the source acceleration voltage and the collision cell was set to 1 kV and argon was used as the collision gas.

## Supplementary Materials

Supplementary materials can be found at www.mdpi.com/1422-0067/17/8/1309/s1.

## Abbreviations

BBB	Blood-brain barrier
BLECs	Brain-like endothelial cells
BM	Brain metastases
Cer	Ceramide
ECs	Endothelial cells
GFP	Green fluorescent protein
GSLs	Glycosphingolipids
HUVECs	Human umbilical vein endothelial cells
LY	Lucifer Yellow
MALDI-TOF	Matrix assisted laser desorption-ionization time-of-flight
MS	Mass Spectrometry
NVU	Neurovascular unit
TACA	Tumor-associated carbohydrate antigen
