# Supplementary material for: ST6GALNAC5 Expression Decreases the Interactions between Breast Cancer Cells and the Human Blood-Brain Barrier
Source: Int J Mol Sci. 2016 Aug 11;17(8):1309. doi: 10.3390/ijms17081309 (PMC5000706; doi:10.3390/ijms17081309)
Supplement: Supplementary file 1 [file ijms-17-01309-s001.pdf]

Int. J. Mol. Sci. **2016**, *17*, 1309; doi:10.3390/ijms17081309

Supplementary Materials: *ST6GALNAC5* Expression Decreases the Interactions between Breast Cancer Cells and the Human Blood-Brain Barrier

AuroreDrolez, Elodie Vandenhaute, Clément P. Delannoy, Justine H. Dewald, Fabien Gosselet, Romeo Cecchelli, Sylvain Julien, Marie-Pierre Dehouck, Philippe Delannoy and Caroline Mysiorek

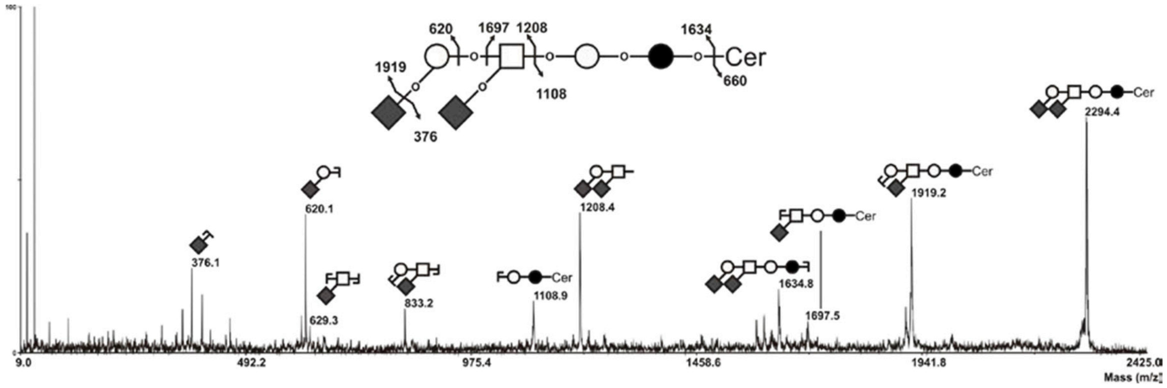

Figure S1. Identification of $G_{D1\alpha}$ ganglioside in the MDA-MB 231 cell population expressing *ST6GALNAC5* cDNA. MS/MS sequencing of permethylated GD1 at *m*/*z* 2295 with ceramide moieties d18:1/C24:0 (Cer**). All fragments are observed as $[M + Na]^+$ adducts. Fragment ions were annotated according to nomenclature of Domon, B. and Costello, C.E., *Biochemistry* **1988**, *27*, 1534–1543. The nature of monosaccharides was deduced from known biosynthesis of gangliosides. ○, Gal; ●, Glc; □, GalNAc; and ◆, Neu5Ac.
